# Electroacupuncture Potentiates Astragaloside IV Cerebral Delivery via P‐Glycoprotein‐Mediated Transcellular Transport: A Novel Blood–Brain Barrier Penetration Strategy for Ischemic Stroke Therapy

**DOI:** 10.1002/brb3.71134

**Published:** 2025-12-17

**Authors:** Ling Ouyang, Xinyi Yang, Jiayue Wu, Bufan Wu, Qidong Huang, Yonglin Chen, Xiaolong Zhang, Yi Cao, Ying Chen, Yirong Yang, Jingjing Zhang, Xiaobei Hao, Shengfeng Lu, Xinyue Jing, Shuping Fu

**Affiliations:** ^1^ Key Laboratory of Acupuncture and Medicine Research of Ministry of Education Nanjing University of Chinese Medicine Nanjing China; ^2^ Traditional Chinese Medicine Hospital of Ruichang Jiujiang China; ^3^ Department of Nutrition and Food Hygiene, School of Public Health Hebei Medical University Shijiazhuang China; ^4^ School of Pharmacy China Pharmaceutical University Nanjing China; ^5^ School of Nursing Nanjing University of Chinese Medicine Nanjing China; ^6^ School of Elderly Care Services and Management Nanjing University of Chinese Medicine Nanjing China

**Keywords:** astragaloside IV, electroacupuncture, ischemic stroke, P‐glycoprotein

## Abstract

**Background:**

Astragaloside IV (AS‐IV) exhibits therapeutic potential in central nervous system (CNS) disorders. However, its brain bioavailability is restricted by P‐glycoprotein (P‐gp)‐mediated efflux at the blood–brain barrier (BBB). This study aims to investigate whether electroacupuncture (EA) can enhance the cerebral delivery of AS‐IV by inhibiting nuclear factor‐kappa B (NF‐κB) nuclear translocation to downregulate P‐gp expression.

**Methods:**

The permeability of the BBB was assessed using Evans blue (EB) staining and transmission electron microscopy. The intracerebral concentration of AS‐IV was quantified by liquid chromatography–tandem mass spectrometry (LC–MS/MS). The therapeutic efficacy of AS‐IV in combination with EA was evaluated using triphenyltetrazolium chloride (TTC) staining and neurological function assessments. mRNA and protein expression levels of relevant factors were analyzed through real‐time quantitative polymerase chain reaction (RT‐qPCR), western blot, and immunofluorescence.

**Results:**

In normal mice, EA effectively increases the levels of EB and AS‐IV in brain tissue. The combination of EA and AS‐IV significantly activates the Wnt/β‐catenin signaling pathway and promotes the expression of zonula occludens protein (ZO)‐1 and Claudin‐5. Furthermore, EA inhibits NF‐κB nuclear translocation and mitigates AS‐IV‐induced upregulation of P‐gp expression. Notably, EA significantly elevates the AS‐IV content in ischemic brain tissue. The combination therapy demonstrates enhanced therapeutic effects in ischemic rats, including reductions in neurological function scores, infarct size, and pro‐inflammatory factor levels, along with increased expression of anti‐inflammatory factors. Additionally, this combination significantly inhibits NF‐κB nuclear translocation and reduces P‐gp expression in the brain tissue of ischemic stroke rats.

**Conclusion:**

These findings indicate that EA enhances the brain uptake and neuroprotective effects of AS‐IV, irrespective of BBB integrity. The underlying mechanism involves P‐gp‐mediated transcellular transport rather than the paracellular pathway.

## Graphical abstract

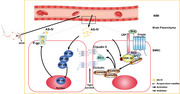

**Abbreviations**
AR: Astragali Radix
AS‐IV: astragaloside IV
BBB: blood‐brain barrier
BMECs: brain microvascular endothelial cells
CNS: central nervous system
EA: electroacupuncture
EAE: experimental autoimmune encephalomyelitis
EB: Evans blue
EBST: elevated body swing test
ECs: endothelial cells
ELISA: Enzyme‐Linked Immunosorbent Assay
FUS: focused ultrasound
GV20: Baihui
I/R: ischemia‐reperfusion
IL‐1β: interleukin‐1β
LC‐MS/MS: liquid chromatography‐tandem mass spectrometry
MB: microbubbles
MCAO: middle cerebral arterial occlusion
MMP‐9: Matrix metalloproteinase‐9
NF‐κB: Nuclear factor‐kappa B
PBS: phosphate‐buffered saline
P‐gp: P‐glycoprotein
p‐GSK‐3β: phosphorylated glycogen synthase kinase‐3β
RT‐qPCR: real‐time quantitative polymerase chain reaction
TJs: tight junctions
TNF‐α: tumor necrosis factor‐α
TTC: triphenyltetrazolium chloride
ZO‐1: zonula occludens protein‐1

## 1 Introduction

Modern medical research has shown that Astragaloside IV (AS‐IV), the principal active constituent of Astragali Radix (AR, Huangqi in Chinese), exhibits diverse therapeutic properties, including anti‐oxidative stress, anti‐inflammatory effects, anti‐apoptosis, and calcium balance. AS‐IV is extensively employed in China for the prevention and treatment of central nervous system (CNS) diseases (Ou et al. 2023; Yang et al. 2023; Zhang, Wu, et al. 2020). Research indicates that AS‐IV significantly delays neuronal aging and effectively treats conditions, such as ischemic stroke, autoimmune encephalomyelitis, Alzheimer's disease, and Parkinson's disease (Zaman et al. 2022; Yin et al. 2020; Zhang et al. 2019; He et al. 2023; Xia et al. 2020). However, studies on its pharmacokinetics reveal that AS‐IV primarily accumulates in the lungs, kidneys, liver, and spleen, with limited brain distribution. This restricted distribution is attributed to its poor lipid solubility and relatively large molecular weight, which hinder its ability to penetrate the blood–brain barrier (BBB) and fully exert its neuropharmacological effects. Enhancing the delivery of AS‐IV into brain tissue to harness its neuroprotective properties remains a critical challenge that limits its therapeutic efficacy (Yang et al. 2023; Wang, Shi, et al. 2022; Chang et al. 2012).

The BBB is primarily composed of brain capillary endothelial cells (ECs) and their tight junctions (TJs), along with perivascular cells, the extracellular matrix, and astrocytes, all of which collectively contribute to maintaining its characteristic low permeability (Salman et al. 2020; Han 2021). Under physiological conditions, the BBB protects the brain by preventing harmful substances from entering and facilitating the removal of metabolic waste, thereby maintaining a stable microenvironment. However, this protective mechanism restricts nearly 100% of large molecular drugs and over 98% of small molecular drugs from crossing the barrier, impeding their pharmacological efficacy (Pardridge 2005). Transport across the endothelial layer occurs via two distinct pathways: paracellular and transcellular. The TJs between ECs form a structural barrier that determines paracellular permeability. The TJ protein zona occludens‐1 (ZO‐1) connects transmembrane proteins, such as occludin and claudins, to the actin cytoskeleton. Upon activation, ZO‐1 contracts, increasing barrier resistance and sealing the intercellular spaces to prevent solute diffusion through the paracellular route (Zhang et al. 2022). In addition, transporters expressed on brain microvascular endothelial cells (BMECs) act as a functional barrier for the BBB, significantly influencing its overall function. P‐glycoprotein (P‐gp), an ATP‐dependent efflux pump, mainly distributed on the luminal membranes of BMECs, extrudes intracellular substances back into the bloodstream, utilizing ATP as an energy source (Zuhl et al. 2014). In a study using an experimental autoimmune encephalomyelitis (EAE) model, AS‐IV administration was found to induce P‐gp expression, which corresponded with decreased levels of AS‐IV in the brain (Zhang et al. 2019). Therefore, the upregulation of P‐gp may be an important factor in preventing the drug from reaching therapeutic levels in tissues, resulting in limited neuroprotection.

Lots of studies have aimed to overcome the BBB to enable CNS drug delivery, employing advanced technologies and pharmaceuticals. Techniques, such as focused ultrasound (FUS) combined with microbubbles (MB), have been shown to transiently and reversibly enhance BBB permeability. However, clinical applications remain limited due to their inherent complexity (Yang et al. 2021). Borneol has emerged as an effective enhancer of BBB penetration (Zheng et al. 2018). Natural borneol is expensive and not easy to obtain, whereas synthetic borneol necessitates chemically modified to reduce toxicity and optimize its permeabilization effect (Song et al. 2018). Furthermore, P‐gp inhibitors are not viable for clinical applications due to some adverse events (Mechetner and Roninson 1992; Yu et al. 2016; Dong et al. 2020; Chen et al. 2016; Matzneller et al. 2018).

Electroacupuncture (EA), a therapy that merges traditional acupuncture with modern electrotherapy, is widely practiced due to its simplicity, efficacy, and minimal side effects (Liu et al. 2017; Mao et al. 2021). Baihui (GV20), a pivotal acupoint in traditional Chinese medicine, is anatomically situated at the vertex of the human head along the Governor Vessel meridian. Clinically integrated into therapeutic protocols for CNS disorders such as stroke, GV20 demonstrates significant neuromodulatory capacity through its established role in cerebral hemodynamic regulation and neurofunctional modulation (Ren et al. 2024). EA at GV20 has been demonstrated to modulate BBB permeability in a stimulus‐dependent and transient manner (Zhang et al. 2018; Zhang, Gong, et al. 2020). Thus, our study highlights the potential of EA in facilitating the passage of AS‐IV across the BBB into the brain and elucidates the underlying mechanism, aiming to provide a novel and valuable approach to increase the intracerebral drugs content for the treatment of CNS disease.

## 2 Materials and Methods

### 2.1 Animals

Adult male specific pathogen‐free BALB/c mice (6–8 weeks old, 20–24 g) and Sprague‐Dawley rats (7–8 weeks old, 260–290 g) from the Experimental Animal Center of Nanjing University of Chinese Medicine were used in this study. Animals were housed under a 12‐h light/dark cycle with unrestricted access to food and water. The researchers performed the study following the guidelines of the Care and Use of Laboratory Animals published by the National Institutes of Health and approved by the Institutional Animal Care and Use Committee of the Nanjing University of Chinese Medicine (animal license: SCXK(HU) 2022‐0004).

### 2.2 EA and Pharmacological Treatment

#### 2.2.1 Part 1

This study aims to observe the degree of BBB opening and the content of AS‐IV transported across the BBB under different durations of EA stimulation in normal mice.

##### 2.2.1.1 Experiment 1

**FIGURE 1:**
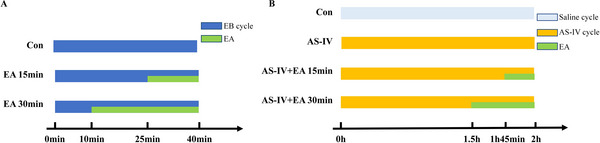
Experimental grouping and intervention in normal mice. (A) Mice injected with EB were divided into Con, EA 15 min and EA 30 min groups to observe the degree of BBB opening under different durations of EA stimulation. (B) Mice injected with AS‐IV were divided into Con, AS‐IV, AS‐IV + EA 15 min, and AS‐IV + EA 30 min groups to investigate the transport of AS‐IV across the BBB under different durations of EA stimulation. AS‐IV, astragaloside IV; EA, electroacupuncture; EB, Evans blue.

Mice were randomly divided into the Con group, EA 15 min group and EA 30 min group. All mice were injected with a 0.4% Evans blue (EB) (Sigma, India) solution via the tail vein (10 mL/kg) and allowed to circulate throughout the brain for 40 min before sacrificing. In the EA groups, two acupuncture needles (Beijing Zhongyan Taihe Medical Instrument Co. Ltd., China) were inserted into the GV20, located at the intersection of the sagittal midline and the line linking the two ears. The needles were then connected to Han's EA instrument (Nanjing Jisheng Medical Technology, Nanjing, China) and underwent EA stimulation at 3 mA, 2/100 Hz for 15 and 30 min, following EB injection for 25 and 10 min, respectively (Figure 1A).

##### 2.2.1.2 Experiment 2

Mice were randomly divided into the Con group, AS‐IV group, AS‐IV + EA 15 min group, and AS‐IV + EA 30 min group. No treatment for the Con group, and other mice were injected with 8 mg/kg AS‐IV (purity >98%, Solarbio, China) via the tail vein and allowed to circulate throughout the brain for 2 h before sacrificing. The EA groups underwent EA stimulation at 3 mA, 2/100 Hz for 15 and 30 min, following AS‐IV injection for 1 h 45 min and 1 h 30 min, respectively (Figure 1B).

#### 2.2.2 Part 2

**FIGURE 2:**
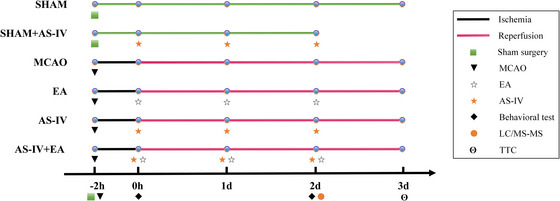
Experimental grouping and interventions in MCAO rats. Rats were divided into the SHAM, SHAM + AS‐IV, MCAO, EA, AS‐IV, and AS‐IV + EA groups to evaluate the therapeutic effect of EA combined with AS‐IV on ischemic brain injury. AS‐IV, astragaloside IV; EA, electroacupuncture; LC–MS/MS, liquid chromatography‐tandem mass spectrometry; MCAO, middle cerebral arterial occlusion; TTC, triphenyltetrazolium chloride.

This study aimed to investigate the therapeutic effect of AS‐IV combined with EA on rats with middle cerebral arterial occlusion (MCAO). A 30‐min EA treatment duration was selected on the basis of the results from Part 1. Rats were randomly divided into six groups: SHAM, SHAM + AS‐IV, MCAO, EA, AS‐IV, and AS‐IV + EA groups. The experimental groups underwent treatment once daily for a total of three times. Immediately after reperfusion, the SHAM + AS‐IV, AS‐IV, and AS‐IV + EA groups received an injection of AS‐IV (16 mg/kg). At 1 h 30 min post‐reperfusion, the EA and AS‐IV + EA groups underwent EA therapy at 3 mA, 2/100 Hz for 30 min (Figure 2).

### 2.3 BBB Permeability Assay

BBB permeability was evaluated by detecting the content of EB. Briefly, EB was administered to mice via the tail vein 40 min before euthanasia. The animals were perfused with pre‐cooled phosphate‐buffered saline (PBS). The left hemisphere of the brain was then quickly extracted on ice and weighed. The tissues were homogenized in formamide solution and subjected to ice‐cold sonication for 30 min, followed by centrifugation at 10,000 *g* for 20 min at 4°C. The supernatants were obtained and the absorbance value was measured at 620 nm wavelength using multifunctional microplate reader (BioTek, USA). The EB content in the extracts was calculated using an EB standard curve and quantified in micrograms per milligram of brain tissue.

Then, in order to observe the distribution of EB in the brain, frozen brain slices with a thickness of 25 µm were prepared. Following incubation with DAPI (Absin, China) at room temperature for 8 min, a full scan of the slice was performed through Thunder Imager Model Organism (Leica Microsystems Srl, Buccinasco, Italy).

### 2.4 Focal Cerebral Ischemia and Neurological Function Scores

All rats were fasted and water‐free the night before surgery. MCAO surgery was performed according to the method of Longa et al. (Longa et al. 1989). After rapid anesthesia of rats with 5% isoflurane (RWD Life Science Co. Ltd., China), the left common carotid artery, external carotid artery, and internal carotid artery were exposed under 2% isoflurane maintenance anesthesia. The external carotid artery was ligated, and a monofilament nylon suture (diameter 0.26 mm) was inserted approximately 18–20 mm from the external carotid artery into the internal carotid artery to block the origin of the middle cerebral artery. Rats in the sham group performed the same surgical operation but without the suture insertion. The rats were placed on a heating pad set to 37°C to maintain body temperature throughout the process.

### 2.5 Neurobehavioral Testing

After 2 h of occlusion, all MCAO rats were neurologically assessed according to Bederson's method (Bederson et al. 1986). Rats scoring 2–3 were included in the experiment, and the suture was removed to initiate reperfusion. All ischemia‐reperfusion (I/R) rats underwent neurological function scoring again 48 h after reperfusion.

The elevated body swing test (EBST) was conducted according to the method described by Wang, Yang, et al. (2022). Briefly, rats were lifted by their tails, and the number of right and left turns was recorded. A total of 20 trials were performed, and the results were calculated as the number of right swings/total number. EBST was conducted with the assistance of an investigator who was blinded to the experimental groups.

### 2.6 Triphenyltetrazolium Chloride (TTC) Staining

After 3 days of reperfusion, the rats were sacrificed in deep anesthesia and the whole brain was extracted and frozen at −20°C. Then, the brain was cut into five slices along the coronal plane equally, and the brain slices were immersed in 2% TTC (Sigma, USA) staining solution and incubated at 37°C for 20 min. Images were captured, and ImageJ (V.1.8.0, USA) was used to calculate the infarct area (%) defined as (total infarcted area/total area) × 100%.

### 2.7 Analysis of AS‐IV Concentration in Brain

Mice were injected with AS‐IV through the tail vein and sacrificed after a 2‐h circulation. Rats were sacrificed 2 h after the third AS‐IV injection. Brains were quickly removed on ice. The left hemispheres were weighed and homogenized in 1 mL of 0.9% saline using an ultrasonic homogenizer. An aliquot of 180 µL of homogenate was taken, and 20 µL of internal standard digoxin was added. After adding 600 µL of methanol and vortexing for 5 min, the mixture was centrifuged at 12,000 rpm for 12 min at 4°C. The supernatant was separated and evaporated under a stream of nitrogen at 37°C (CentriVap Concentrator, Labconco, Germany). The residue was reconstituted with 200 µL of 50% methanol, vortexed for 5 min, sonicated for 20 min, and then centrifuged at 12,000 rpm for 10 min at 4°C. The supernatant was collected and then analyzed by liquid chromatography‐tandem mass spectrometry (LC–MS/MS).

The LC–MS/MS analysis of AS‐IV was conducted using an LC–MS/MS system consisting of Exion LC (AB Sciex, Japan) and Triple Quad 6500+ (AB Sciex, Singapore). Chromatographic separation utilized a C18 1.7 µm 2.1 × 100 mm column (Waters Acquity, Ireland) and 0.1% formic acid (Thermo Fisher, China) in distilled water and acetonitrile (Merck Millipore, Germany) as mobile phases. The flow rate was 0.3 mL/min with column temperature at 30°C. The autosampler was kept at 5°C during the analysis. Tables 1 and 2 display the gradient elution profile for mice and rats, with a total run time of 8 or 10 min.

**TABLE 1:** Gradient conditions for astragaloside IV (AS‐IV) in mice.

Time (h)	0.1% FA in DW (%)	ACN (%)	Flow rate (mL/min)
0	90	10	0.3
0.5	90	10	0.3
2.5	50	50	0.3
5.5	0	100	0.3
6.0	0	100	0.3
7.0	90	10	0.3
8.0	90	10	0.3

Abbreviations: ACN, acetonitrile; DW, distilled water; FA, formic acid.

**TABLE 2:** Gradient conditions for astragaloside IV (AS‐IV) in rats.

Time (h)	0.1% FA in DW (%)	ACN (%)	Flow rate (mL/min)
0	90	10	0.3
0.5	90	10	0.3
2.5	50	50	0.3
5.5	0	100	0.3
8.0	0	100	0.3
8.1	90	10	0.3
10.0	90	10	0.3

Abbreviations: ACN, acetonitrile; DW, distilled water; FA, formic acid.

The electrospray ionization (ESI) source was operated in the positive mode. The parameters for MS were set as follows: ion source gas 1, 55 psi; ion source gas 2, 55 psi; ion source temperature, 350°C; voltage, 5500 V; curtain gas, 45 psi. Declustering potential 100.0 V, collision energy 60.0 V, and collision cell exit potential 6.0 V, for AS‐IV; declustering potential 60.0 V, collision energy 71.0 V, and collision cell exit potential 6.0 V for IS. The selected *m/z* ions for MRM analysis were 803.4 → 387.0 for AS‐IV and 807.4 → 627.3 for Digoxin. Sciex OS 2.1.6 Analytics (Sciex) was used to process the mass spectrometric data.

### 2.8 Immunofluorescence Staining

The brains of mice and rats were fixed overnight and subsequently dehydrated in sucrose until the tissue sank to the bottom. Next, the brain tissue was sectioned to a thickness of 20 µm and washed in PBS containing 0.3% Tween. It was then blocked at 37°C for 1.5 h with 5% goat serum (Beyotime, China) containing 0.05% Triton (Biosharp, China). The sections were incubated overnight at 4°C with primary antibodies, including P‐gp (Abcam, Cam‐bridge, UK) and nuclear factor‐kappa B (NF‐κB) (Cell Signaling, USA). The sections were then incubated with secondary antibodies, Alexa Fluor 488 (Thermo Fisher Scientific, Waltham, MA, USA), for 1 h at 37°C, followed by staining the nuclei with DAPI for 10 min at room temperature. All sections were overlaid with a coverslip and viewed under a Thunder Imager Model Organism (Leica Microsystems Srl, Buccinasco, Italy). For quantitative analysis, the percentage of P‐gp‐positive and NF‐κB‐positive cells was determined in four randomly selected fields from the mouse cortex and rat ischemic penumbra, respectively.

### 2.9 Western Blot

The brain tissues of mice and rats were homogenized in RIPA buffer (Beyotime, China) containing protease inhibitors (Sigma, Germany) and protein phosphatase inhibitors (Beyotime). Then centrifuged at 4°C for 20 min at 12,000 rpm, and the supernatants were stored at −80°C. Nuclear extracts were prepared using a nuclear and cytoplasmic protein extraction kit (Beyotime), following the manufacturer's instructions. The protein concentration of each sample was quantified using the bicinchoninic acid assay (Yeasen, China) and normalized. The total protein (20 µg for brain samples) was isolated by electrophoresis and then transferred to polyvinylidene fluoride (PVDF) membranes (Millipore, Burlington, MA, USA). The membranes were blocked with 5% BSA for 2 h and incubated overnight at 4°C with the corresponding primary antibodies as follows: anti‐ZO‐1 (21773‐1‐AP, 1:2000, proteintech, USA), anti‐P‐gp (ab168337, 1:6000 or 1:7500 for rat, Abcam, Cam‐bridge, UK), anti‐β‐catenin (8480S, 1:1000 CST, USA), anti‐GSK‐3β (12456S, 1:1000, CST), anti‐phosphorylated glycogen synthase kinase (*p*‐GSK)‐3β (5558S, 1:1000, CST), anti‐NF‐κB (8242S, 1:1000 CST), anti‐Claudin‐5 (A10207, 1:2000, ABclonal, China), and anti‐Tubulin (FD0064, 1:8000, FDbio, China), anti‐β‐actin (AC026, 1:100000, ABclonal), anti‐Histone H3 (4620S, 1:1000 CST). The membranes were then incubated with HRP‐conjugated goat anti‐rabbit/mouse (H + L) secondary antibody (7074S, 1:3500, CST)/(L3032, 1:45000, SAB, USA) for 1 h at room temperature. Finally, they were visualized with supersensitive chemiluminescence reagent (Abbkine, China) and Fusion FX7 Edge system (Vilber, France). Anti‐β‐actin or Tubulin was used to control equal loading and protein quality. The band intensity was quantified by the EvolutionCapt software.

### 2.10 Real‐Time Quantitative Polymerase Chain Reaction (RT‐qPCR)

Total RNA was extracted from the ischemic brain of rats using Trizol (Vazyme, China) and then reverse transcribed into complementary DNA (cDNA) using HiScript Q RT SuperMix for qPCR (Vazyme, China). The cDNA was subsequently used for quantitative RT‐PCR by Genious 2× SYBR Green Fast qPCR Mix (ABclonal) on a QuantStudio 7 system (Applied Biosystems, USA) according to the manufacturer's instruction. Data were analyzed by the ΔΔ*CT* method. The primer sequences were shown in Table 3.

**TABLE 3:** Primer sequences for real‐time quantitative polymerase chain reaction (RT‐qPCR) analysis.

Gene	Primer sequences 5′ → 3′			
	Forward	Reverse	Annealing Temperature (°C)	Product Length (bp)
TNF‐α	GCTACGGGCTTGTCACTC	CCACGCTCTTCTGTCTACTG	60	136
IL‐1β	AGGTCGTCATCATCCCAC	TTCAAATCTCACAGCAGCAT	60	190
IL‐10	TGGCTCAGCACTGCTATGTT	CTGGGAAGTGGGTGCAGTTA	60	98
GAPDH	GGCACAGTCAAGGCTGAGAATG	ATGGTGGTGAAGACGCCAGTA	60	143

Abbreviations: IL‐1β: interleukin‐1β; IL‐10: interleukin‐10; TNF‐α: tumor necrosis factor‐α.

### 2.11 Enzyme‐Linked Immunosorbent Assay (ELISA)

To quantify the levels of tumor necrosis factor‐α (TNF‐α) and interleukin‐1β (IL‐1β), brain tissues were homogenized in 0.9% saline and subsequently centrifuged at 3000 *g* for 10 min to collect the supernatant. The supernatants were analyzed using specific ELISA kits (Jiangsu Meimian Biotechnology Co. Ltd., China). The assay procedures were performed in accordance with the manufacturers’ protocols.

### 2.12 Statistical Analysis

Data were analyzed using one‐way ANOVA and expressed as mean ± SD, performed with Prism 9.0.0. One‐way ANOVA, followed by the Dunnett T3 post hoc test, was used for comparison among groups. A *p* < 0.05 was considered significantly different.

## 3 Results

### 3.1 EA Can Significantly Increase BBB Permeability and Intracerebral AS‐IV Content

**FIGURE 3:**
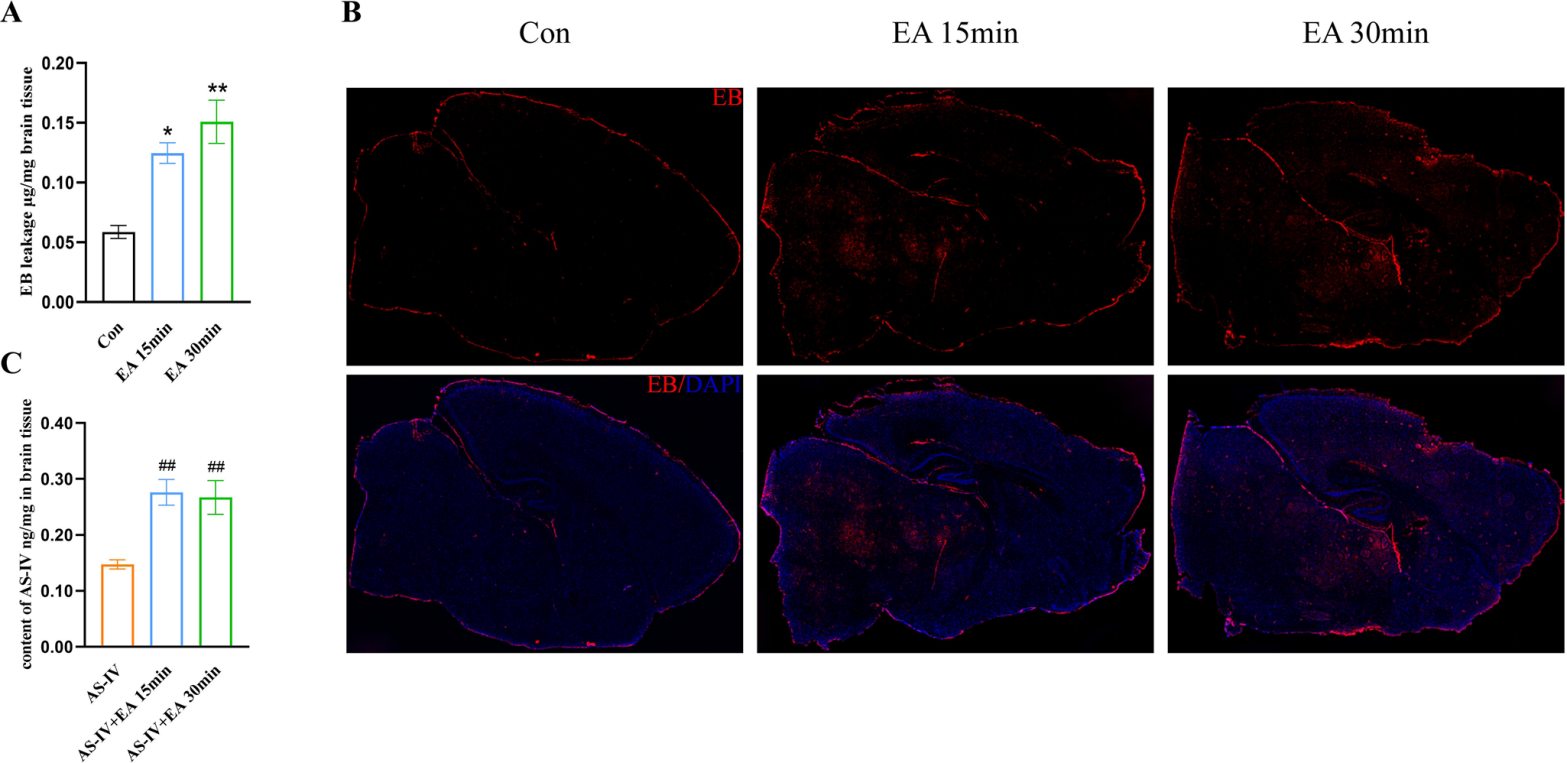
Content of EB and AS‐IV at different durations of EA intervention in the brain. (A) Quantification of Evans blue leakage in the brain (*n* = 6). (B) Representative images of EB distribution in sagittal brain slices (*n* = 3). (C) Quantification of AS‐IV Brain Intake (*n* = 6). Data are expressed in mean  ± SD, ^*^ *p* < 0.05, ^**^ *p* < 0.01 versus Con group; ^#^ *p* < 0.05, ^##^ *p* < 0.01 versus AS‐IV group. AS‐IV, astragaloside IV; EA, electroacupuncture; EB, Evans blue.

EB extravasation assay was used to detect the BBB permeability. It showed that EA stimulation for 15 and 30 min significantly promoted EB penetration into the brain compared with the untreated Con group (Figure 3A). Fluorescent imaging of EB (Figure 3B) displayed substantial red fluorescence in the brain parenchyma of the EA 15 and EA 30 min groups, indicating a reduction in BBB tightness.

To further investigate whether EA facilitates AS‐IV transport across the BBB, we applied the same EA treatment to normal mice subjected to AS‐IV. Results showed that AS‐IV content in the AS‐IV + EA 15 and AS‐IV + EA 30 min groups was significantly elevated compared to the AS‐IV group (Figure 3C). On the basis of the results of the intracerebral content of EB and AS‐IV, we further investigated the mechanisms by which EA facilitates the entry of AS‐IV into the brain.

### 3.2 EA Combined With AS‐IV Increase the Expression of BBB Structural Barrier‐Related Proteins

**FIGURE 4:**
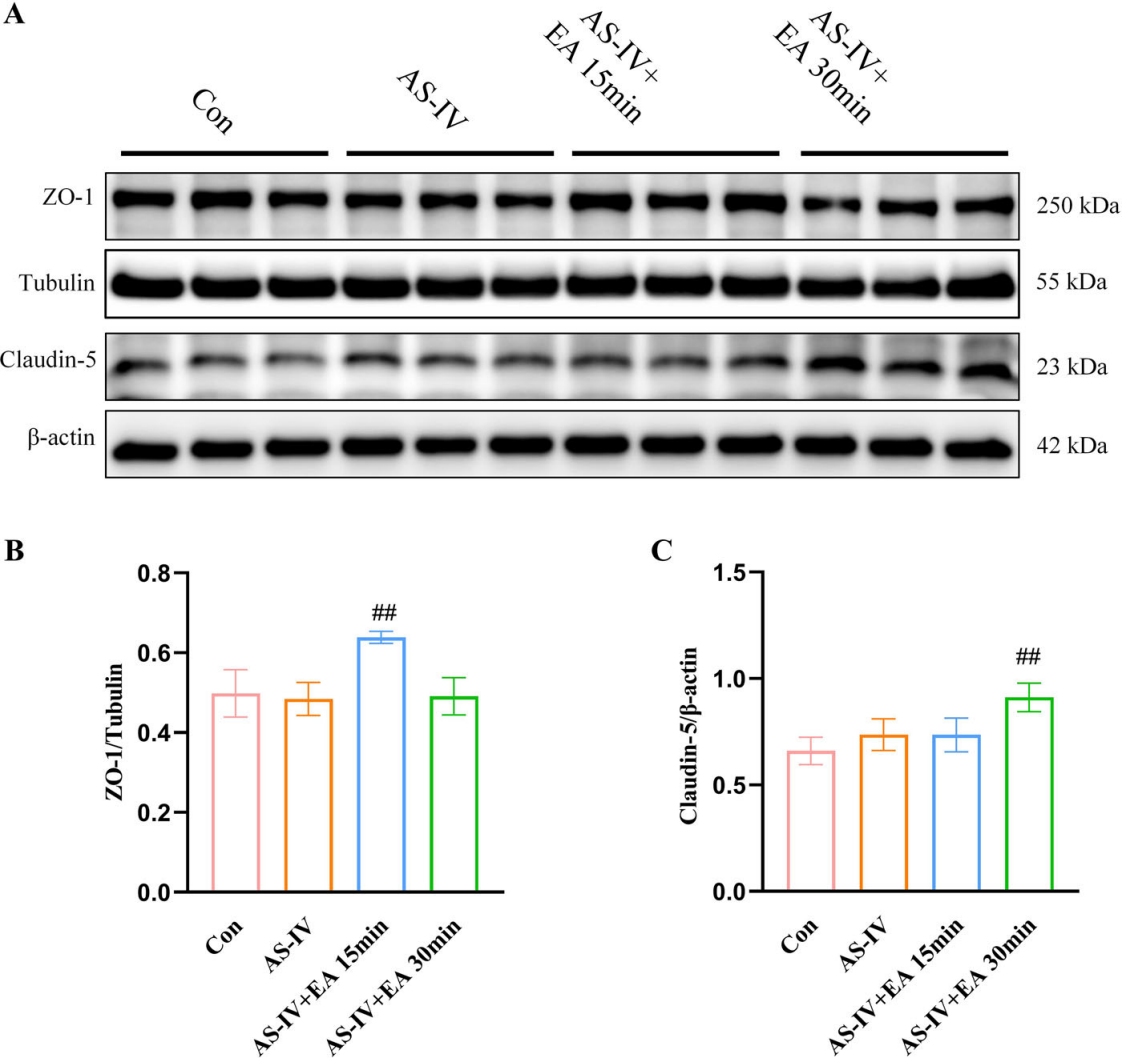
Changes in Claudin‐5 and ZO‐1 protein levels after EA combined with AS‐IV intervention. (A) Representative western blot images of ZO‐1 and Claudin‐5. (B and C) Quantification of ZO‐1 and Claudin‐5 protein levels. Data are expressed in mean ± SD, ^##^ *p* < 0.05 versus AS‐IV group. AS‐IV, astragaloside IV; EA, electroacupuncture.

ZO‐1 and Claudin‐5 are essential for maintaining the integrity of the BBB. Hence, this study first examined the impact of EA combined with AS‐IV on the expression of TJs. Western blot results showed that AS‐IV alone did not change the expression levels of ZO‐1 and Claudin‐5. However, when combined with EA, we found significant increases in the expression of endothelial TJs. Specifically, the expression levels of ZO‐1 and Claudin‐5 were markedly elevated in AS‐IV + EA 15 min group and AS‐IV + EA 30 min group, respectively (Figure 4A–C). These findings indicated that EA increasing the brain penetration of AS‐IV through mechanisms distinct from the paracellular pathway.

### 3.3 EA Inhibits AS‐IV‐Induced Inactivation of the Wnt/β‐Catenin Signaling Pathway

**FIGURE 5:**
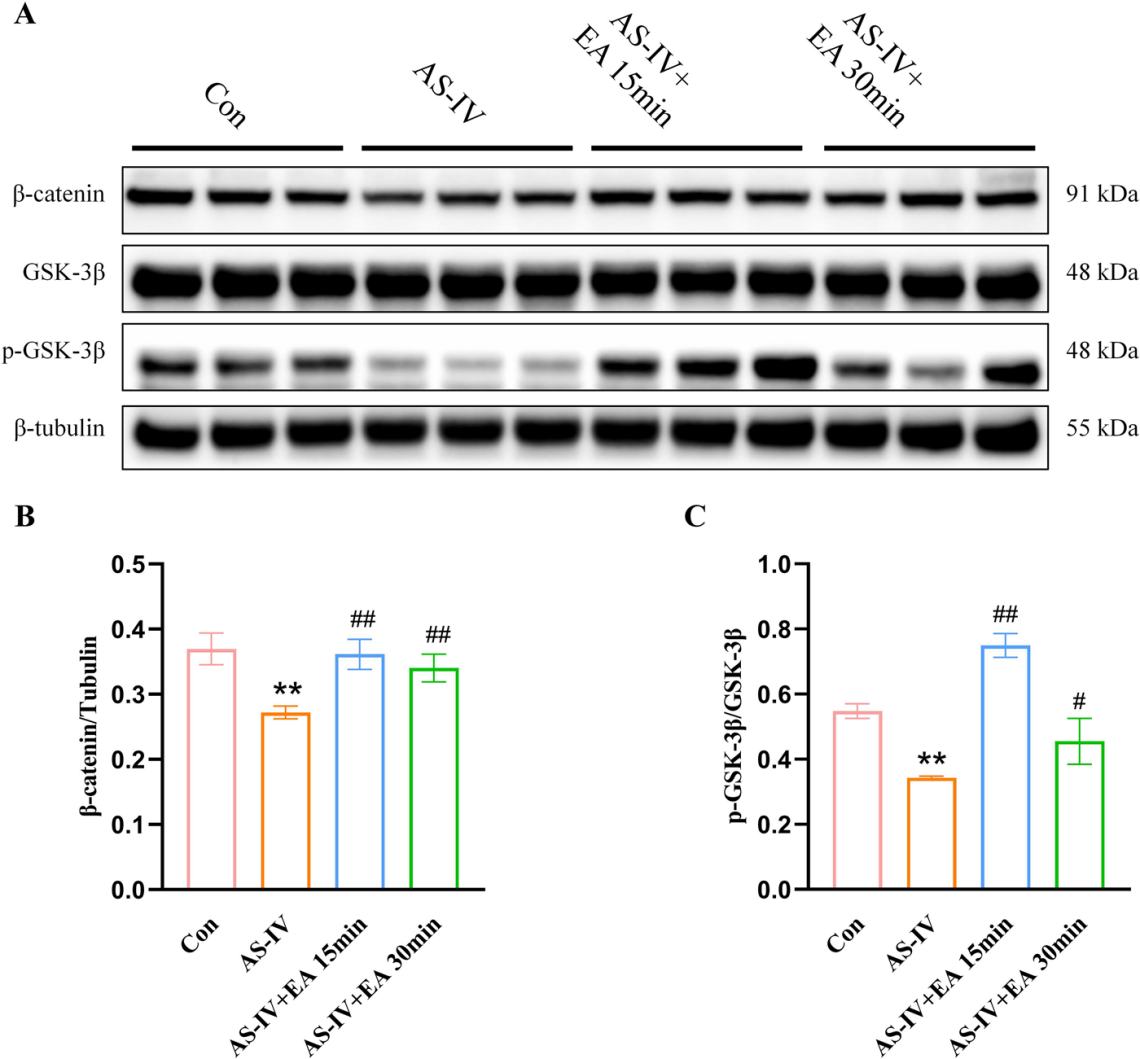
Wnt/β‐catenin signaling pathways were activated by EA combined with AS‐IV. (A) Representative western blot images of β‐catenin, GSK‐3β and *p*‐GSK‐3β. (B and C) β‐catenin, GSK‐3β and *p*‐GSK‐3β protein levels. Values are mean ± SD, ^**^ *p* < 0.01 versus Con group; ^##^ *p* < 0.01 versus AS‐IV group. AS‐IV, astragaloside IV; EA, electroacupuncture; *p*‐GSK, phosphorylated glycogen synthase kinase.

Previous studies have shown that the Wnt/β‐catenin signaling pathway regulates TJs after ischemic damage (Zhao et al. 2020; Ye et al. 2019). We sought to investigate whether this pathway also regulates TJs in normal mice. Western blot analysis showed that, compared to the Con group, the expression levels of both β‐catenin and *p*‐GSK‐3β were decreased in the AS‐IV group. In contrast, expression levels increased in the AS‐IV + EA 15 and AS‐IV + EA 30 min groups (Figure 5A–C).

### 3.4 EA Reverses AS‐IV‐Induced Upregulation of P‐gp Expression

**FIGURE 6:**
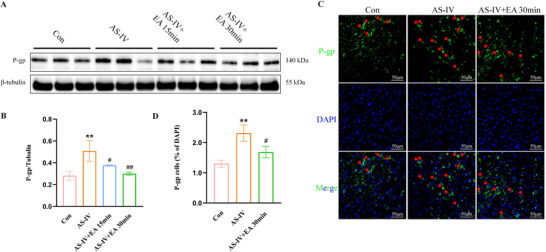
EA reverses AS‐IV‐induced P‐gp expression. (A) Representative western blot images of P‐gp. (B) P‐gp protein levels. (C) Representative immunofluorescence staining (magnification, ×400) of P‐gp (in green) and DAPI (in blue) in mouse brain, indicated by the arrows. (D) Quantitative analysis of P‐gp protein. Scale bar: 50 µm. Values are mean ± SD, ^*^ *p* < 0.05, ^**^ *p* < 0.01 versus Con group; ^#^ *p* < 0.05, ^##^ *p* < 0.01 versus AS‐IV group. AS‐IV, astragaloside IV; EA, electroacupuncture; P‐gp, P‐glycoprotein; *p*‐GSK, phosphorylated glycogen synthase kinase.

P‐gp is an efflux transporter that anchored into the ECs and serves as a functional barrier of BBB, expelling substances from the CNS outward. Our results showed that P‐gp levels in the AS‐IV group were significantly increased compared to the Con group, suggesting that AS‐IV induces P‐gp expression, whereas, as expected, EA combined with AS‐IV reversed this increase in P‐gp levels, with EA administered for 30 min demonstrating enhanced efficacy in reducing AS‐IV‐induced P‐gp expression (Figure 6A,B). Immunofluorescence staining revealed a higher percentage of P‐gp^+^ cells in the AS‐IV group compared to the Con group, indicating that AS‐IV up‐regulated P‐gp expression in BMECs. But this upregulation was reversed by the addition of EA. Therefore, we further explored the mechanism by which EA reduces P‐gp expression (Figure 6C,D).

### 3.5 EA Decreases AS‐IV‐Induced NF‐κB Translocation to the Nucleus

**FIGURE 7:**
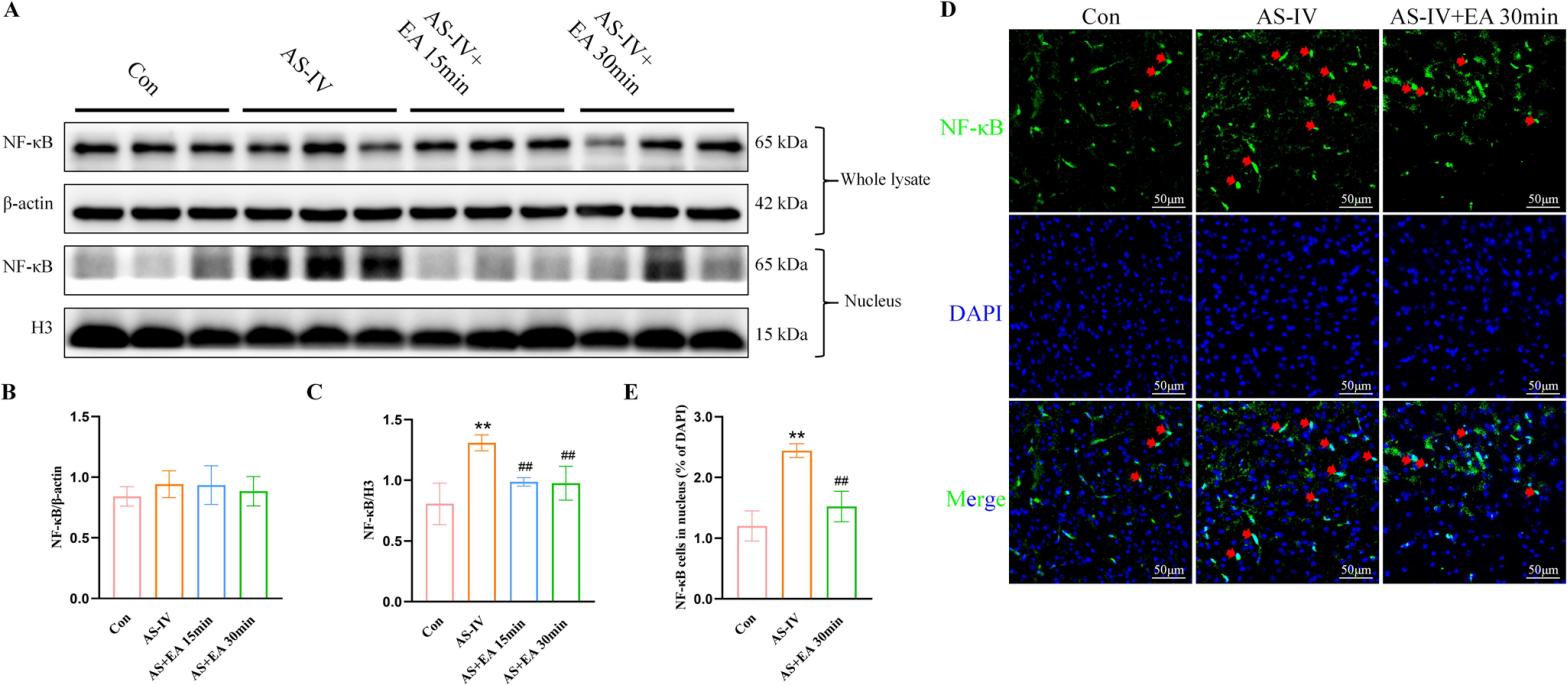
NF‐κB signaling pathways were activated by EA combined with AS‐IV. (A) Representative western blot images of NF‐κB in whole lysate and nuclear fractions. (B) NF‐κB protein levels in whole lysate and (C) NF‐κB protein levels in nucleus. (D) Representative immunofluorescence staining (magnification, ×400) of NF‐κB (in green) and DAPI (in blue) in mouse brain, indicated by the red arrows, and (E) quantitative analysis of NF‐κB protein in nucleus. Scale bar: 50 µm. Values are mean ± SD, ^*^ *p* < 0.05, ^**^ *p* < 0.01 versus Con group; ^##^ *p* < 0.01 versus AS‐IV group. AS‐IV, astragaloside IV; EA, electroacupuncture; NF‐κB, nuclear factor‐kappa B.

NF‐κB activation is associated with the upregulation of P‐gp expression. Thus, the NF‐κB total protein was detected. It showed no significant changes in total NF‐κB protein levels among the groups (Figure 7A,B). However, the expression of nuclear NF‐κB protein increased in the AS‐IV group, whereas it decreased significantly in the AS‐IV + EA 15 and 30 min groups compared to the AS‐IV group (Figure 7A,C). Immunofluorescence staining further confirmed that EA reduced the AS‐IV‐induced translocation of NF‐κB to the nucleus (Figure 7D,E).

### 3.6 EA Enhances Brain Uptake and Neuroprotective Effects of AS‐IV in I/R‐Injured Rat

**FIGURE 8:**
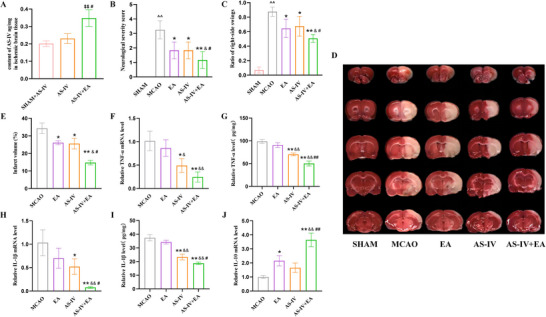
Cerebroprotective effects of EA combined with AS‐IV in I/R rats. (A) Quantification of AS‐IV brain intake (*n* = 6). (B) Neurological severity score (*n* = 12), (C) EBST evaluation (*n* = 7–10), (D) representative images of TTC‐stained brain sections (*n* = 5), and (E) quantification of infarct volume (*n* = 4). (F–J) RT‐qPCR and ELISA analysis of the expression levels of pro‐inflammatory factors TNF‐α and IL‐1β, and anti‐inflammatory factor IL‐10 in the ischemic brain, (*n* = 4). Values are mean ± SD, ^$$^ *p* < 0.01 versus SHAM + AS‐IV group, ^^^^ *p* < 0.05 versus SHAM group, ^*^ *p *< 0.05, ^**^ *p* < 0.01 versus MCAO group, ^&^ *p* < 0.05, ^&&^ *p* < 0.01 versus EA group, ^#^ *p* < 0.05, ^##^ *p* < 0.01 versus AS‐IV group. AS‐IV, astragaloside IV; EA, electroacupuncture; MCAO, middle cerebral arterial occlusion; TNF‐α, tumor necrosis factor‐α.

In order to observe the protective effect of EA combined with AS‐IV on brain I/R injury, rats received EA and/or AS‐IV treatment three times after MCAO. First, we measured the AS‐IV content in the ischemic brains of the rats. The results showed that AS‐IV content in the AS‐IV + EA group was significantly higher than those in the SHAM + AS‐IV and the AS‐IV groups (Figure 8A). Subsequently, we assessed the I/R injury status across these groups. As shown in Figure 8B, rats in MCAO group exhibit severe neurological deficits. Neurological deficits in each treatment group were alleviated compared with the MCAO group, with the AS‐IV + EA group demonstrating significantly lower neurological function scores. Similar results were observed in EBST, where rats treated with AS‐IV + EA had a better balance in swings ratio compared to the other groups (Figure 8C). A significant positive correlation was observed between the Bederson neurological scores and the lateralization index from the EBST, confirming the concordance of these behavioral assessments in quantifying ischemic neurological impairment (Figure S1). TTC staining showed that the volume of cerebral infarction significantly increased in the MCAO group relative to the SHAM group, but the lesion area decreased in treatment groups, among which, the AS‐IV + EA group exhibited the smallest infarct area (Figure 8D,E).

The inflammatory response is a crucial factor influencing the prognosis of ischemic injury. We assessed the expression of inflammatory markers in the infarcted hemisphere of rats across all groups using RT‐qPCR. The results indicated that EA treatment alone did not reduce the mRNA and protein levels of TNF‐α and IL‐1β. However, AS‐IV exhibited significant anti‐inflammatory effects, which were further enhanced when AS‐IV was combined with EA (Figure 8F–I). In terms of the anti‐inflammatory factor interleukin‐10 (IL‐10), EA alone was able to increase its levels, whereas the AS‐IV group showed a trend toward increase, although this was not statistically significant. Notably, the AS‐IV + EA group demonstrated the most pronounced enhancement in IL‐10 levels (Figure 8J).

### 3.7 EA Combined With AS‐IV Increase the Expression of Structural Barrier Proteins in I/R‐Injured Rats

**FIGURE 9:**
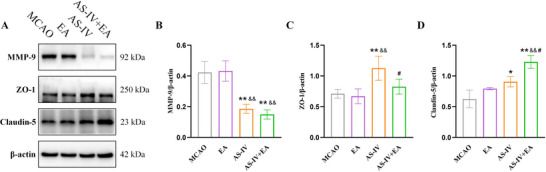
EA combined with AS‐IV affects BBB structural barrier proteins in I/R rats. (A) Representative western blot images of MMP‐9, ZO‐1, and Claudin‐5. (B–D) Quantification of MMP‐9, ZO‐1 and Claudin‐5 protein levels. Values are mean ± SD, ^*^ *p *< 0.05, ^**^ *p* < 0.01 versus MCAO group, ^&&^ *p* < 0.01 versus EA group, ^#^ *p* < 0.05 versus AS‐IV group. AS‐IV, astragaloside IV; EA, electroacupuncture; MCAO, middle cerebral arterial occlusion.

Matrix metalloproteinase (MMP)‐9 is a biomarker associated for BBB disruption. In the EA group, MMP‐9 expression did not significantly differ from that in the MCAO group. However, both the AS‐IV and the AS‐IV + EA groups exhibited significant reduction in MMP‐9 protein levels (Figure 9A,B). Additionally, ZO‐1 expression increased exclusively in the AS‐IV group (Figure 9A,C), whereas Claudin‐5 expression was elevated in both the AS‐IV and AS‐IV + EA groups, with a particularly pronounced increase in the AS‐IV + EA group (Figure 9A,D).

### 3.8 EA Reduces AS‐IV‐Induced P‐gp Expression by Inhibiting NF‐κB Translocation to the Nucleus in I/R‐Injured Rats

**FIGURE 10:**
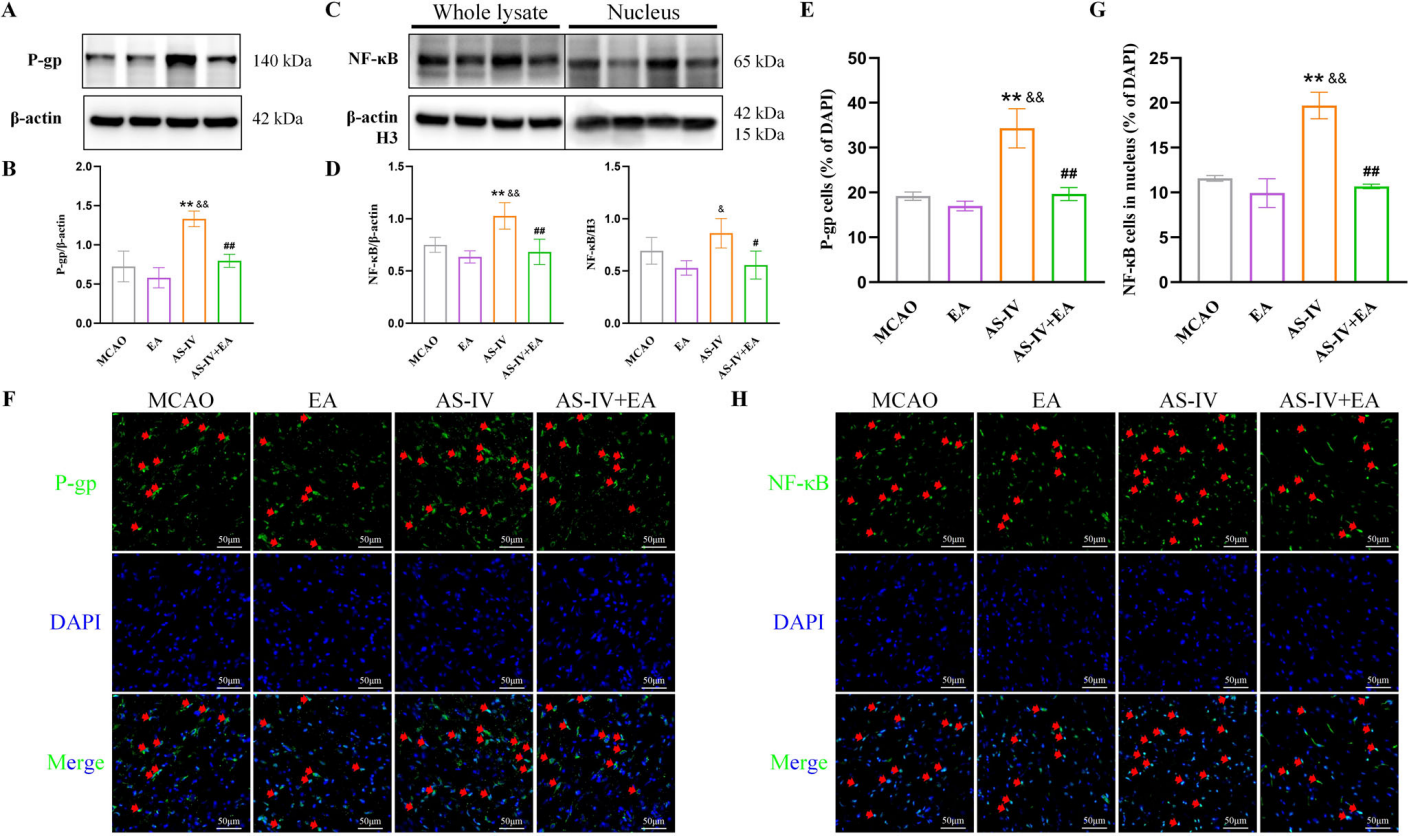
EA combined with AS‐IV affects the BBB functional barrier proteins in I/R rats. (A) Representative western blot images of P‐gp, NF‐κB in whole lysate and NF‐κB in nucleus, (B) P‐gp protein levels, (C) NF‐κB protein levels in whole lysate, and (D) NF‐κB protein levels in nucleus. (F) and (H) Representative immunofluorescence staining (magnification, ×400) of P‐gp, NF‐κB (in green) and DAPI (in blue) in the rat brain, indicated by red arrows. (E and G) Quantitative analysis of P‐gp and intranuclear NF‐κB protein levels. Scale bar: 50 µm. Values are mean ± SD, ^**^ *p* < 0.01 versus MCAO group, ^&^ *p* < 0.05, ^&&^ *p* < 0.01 versus EA group, ^#^ *p* < 0.05, ^##^ *p* < 0.01 versus AS‐IV group. AS‐IV, astragaloside IV; EA, electroacupuncture; MCAO, middle cerebral arterial occlusion; NF‐κB, nuclear factor‐kappa B; P‐gp, P‐glycoprotein.

Analysis of P‐gp and associated pathway proteins revealed that EA alone did not significantly alter P‐gp expression levels in the brains of stroke rats. However, AS‐IV significantly induced P‐gp expression, whereas EA effectively reduced the elevated P‐gp levels induced by AS‐IV (Figure 10A,B). Additionally, AS‐IV increased both total protein and nuclear levels of NF‐κB, whereas EA reversed this effect (Figure 10C,D). Fluorescence experiments corroborated similar results, showing no significant differences in the proportions of P‐gp^+^ and nuclear NF‐κB^+^ cells between the MCAO and EA groups. In contrast, the AS‐IV group showed a significant increase in both P‐gp^+^ and nuclear NF‐κB^+^ cells, whereas the AS‐IV + EA group demonstrated a marked reduction in these populations compared to the AS‐IV group (Figure 10E–H).

## 4 Discussion

Our findings demonstrate that EA enhances BBB permeability, leading to increased intracerebral concentrations of AS‐IV. This effect is mediated through P‐gp‐dependent transcellular transport, irrespective of BBB integrity.

AR is a traditional Chinese herb renowned for its diverse pharmacological properties, including anti‐inflammatory, anti‐tumor, neuroprotective, anti‐diabetic, antioxidant, and immune‐regulatory effects (Yu et al. 2022). Formulas with AR as the main ingredient (such as Buyang Huanwu decoction, and Huangqi Guizhi Wuwu decoction) have been pivotal in the clinical management of stroke (Gao et al. 2021; Shi et al. 2023). AS‐IV, the principal bioactive compound in *Astragalus* species, serves as a critical marker for assessing the quality of AR and its preparations as outlined in the Chinese Pharmacopoeia (2015 version) (Yu et al. 2022; Wang et al. 2017). An experiment shows that AS‐IV has certain permeability in the BBB, and is recognized as a potent neuroactive compound (Stępnik and Kukula‐Koch 2020). Its strong antioxidant and anti‐inflammatory properties render AS‐IV a promising therapeutic agent for neurodegenerative diseases (Zaman et al. 2022; Yin et al. 2020). The protective effect of AS‐IV against LPS‐induced BBB dysfunction may involve the activation of the Nrf2 antioxidant pathway (Li et al. 2018). Moreover, AS‐IV has been shown to maintain BBB integrity during I/R injury by up‐regulating TJs protein expression (Li et al. 2018) and to mitigate ischemic brain injury by downregulating pro‐inflammatory factors while upregulating anti‐inflammatory ones (Yao et al. 2023). Nonetheless, AS‐IV exhibits extremely low bioavailability, because its high molecular weight and poor lipophilicity restricts its ability to cross the BBB and fully exert its central protective effects. This is a common issue shared by most of the neuroprotective drugs. To overcome this limitation, recent research has focused on developing novel delivery strategies, particularly those involving nanocarriers. For instance, β‐asarone modified AS‐IV loaded chitosan nanoparticles (AS‐IV‐βCS‐NP) were designed for enhanced nose‐to‐brain delivery (Zhao et al. 2023). In addition, a liposomal system co‐loaded with AS‐IV and nestifin‐1 (NF‐1) was developed. These liposomes, functionalized with wheat germ agglutinin and leptin (WGA‐Lep‐AS‐IV‐NF‐1‐PS‐liposomes), significantly improved the capacity of AS‐IV to penetrate the BBB (Kuo et al. 2021). Although these advanced strategies successfully optimize the pharmacokinetic profile of natural products and expand their clinical application scope, their widespread adoption is still hindered by challenges related to stability, high cost, and difficulties in scalable manufacturing.

EA combines traditional acupuncture techniques with modern electrical stimulation, emerging as a widely accepted clinical intervention due to its safety and lack of reported side effects (Yin et al. 2022; Zhan et al. 2018). We have demonstrated that EA has therapeutic benefits in the animal models of stroke (Wang, Chen, et al. 2023; Fu et al. 2023), and recent findings suggest that EA can open the BBB without causing adverse effects (Zhang et al. 2018; Zhang, Gong, et al. 2020). The mechanism involved may be by activating the p65‐VEGFA‐TJs pathway and inhibiting the Shh‐Gli1 signaling pathway (Lin et al. 2023; Dai et al. 2025). In the present study, we observed that EA significantly increased the permeability of BBB, as manifested by the increase in the intracerebral content and distribution of EB. Additionally, EA augmented AS‐IV uptake in the brains of normal mice, with no notable difference in AS‐IV levels between different EA intervention durations. These observations prompted further investigation into the mechanisms by which EA enhances BBB permeability and facilitates AS‐IV entry into the brain.

The BBB serves as the primary interface for exchange between the bloodstream and the CNS. The main factors impeding drug passage through the BBB include TJs, efflux, and metabolic systems of the BBB. TJs establish a physical barrier by sealing off paracellular pathways, thereby blocking the passive diffusion of hydrophilic endogenous and exogenous substances. Numerous studies have focused on enhancing drug delivery to the brain to improve the efficacy by modulating TJs expression. FUS‐based BBB opening, despite its promise, remains constrained by its significant dependence on specialized equipment, the need for MB co‐administration, susceptibility to interference (e.g., MRI static fields), potential bio‐safety risks from mechanical disruption, and challenges in parameter optimization and standardization (Perolina et al. 2024; Balzano et al. 2025; Yang et al. 2021; Géraudie et al. 2025; Chen et al. 2022). Borneol, although beneficial as a chemical permeabilizer, lacks target specificity and its use as a primary BBB opening strategy carries risks of non‐selective effects. Borneol's effectiveness depends on co‐administration with therapeutic agents and its mechanism might involve altering membrane fluidity, potentially carrying risks of non‐selective disruption and systemic side effects at higher doses (Li et al. 2024; Zhang et al. 2017). Even hyperosmotic mannitol, although FDA‐approved, has potential side effects, including focal seizures, as it disrupts the BBB by causing EC shrinkage (Rapoport 2001; Schulz et al. 2023). In contrast, EA offers a fundamentally safer and more physiologically oriented approach. Instead of physically or chemically forcing the BBB open, all are achievable with a relatively simple and non‐invasive intervention. This positions EA as a valuable strategy, particularly where sophisticated equipment is unavailable or where the risks associated with physical/chemical BBB disruption are deemed undesirable. In our investigation, the combination of EA and AS‐IV increased the expression of ZO‐1 and Claudin‐5 at 15 and 30 min, respectively. β‐Catenin plays a crucial role in the stability of intercellular junctions and vascular permeability, whereas the canonical Wnt/β‐catenin signaling pathway is essential for brain development and BBB maturation (Jean Leblanc et al. 2019). It has been demonstrated that the Wnt/β‐catenin signaling pathway is associated with the expression of TJs (Zhao et al. 2020). Our results indicate that EA and AS‐IV co‐treatment enhances GSK‐3β phosphorylation and β‐catenin expression, suggesting that this pathway upregulates ZO‐1 and Claudin‐5 expression, aligning with previous studies that indicate protective effects of both EA and AS‐IV on the BBB (Li et al. 2018; Jung et al. 2016). Thus, EA may facilitate AS‐IV entry into the brain through mechanisms distinct from altering the structural integrity of the BBB.

P‐gp is a crucial member of ATP‐driven efflux transporters that act as a functional barrier to the BBB. It exports metabolic waste and limits xenobiotics, including most of the therapeutic drugs, from entering to the brain. A number of P‐gp inhibitors, such as verapamil (Tsuruo et al. 1981; Broxterman et al. 1988), cyclosporin A (Shiraga et al. 2001), valspodar (Bark and Choi 2010), tariquidar (Zhang et al. 2019; Mollazadeh et al. 2018), have been developed, yet their clinical application is limited by toxic side effects and poor selectivity (Schulz et al. 2023). AS‐IV has been identified as a substrate of P‐gp and can induce P‐gp expression, thereby enhancing its efflux activity (Zhang et al. 2016, 2019; Lou et al. 2019; Gao et al. 2020). Thus, in this study, we observed that AS‐IV induced P‐gp expression under physiological conditions, an effect that was reversed by EA treatment. This result indicates that EA can increase the intracerebral AS‐IV content by inhibiting the expression of P‐gp. Furthermore, the promoter region of the human ABCB1 gene contains a transcription factor NF‐κB, whose activation has been linked to the upregulation of P‐gp expression (Chai et al. 2022; Abdin et al. 2021). Our findings reveal that AS‐IV decreases NF‐κB expression, whereas the combination of EA and AS‐IV increases it. Notably, AS‐IV significantly elevated intranuclear NF‐κB levels, which were attenuated by EA. These results indicate that EA may counteract AS‐IV‐induced increases in P‐gp expression by reducing NF‐κB nuclear translocation, thereby promoting higher intracerebral AS‐IV levels.

Previous studies have demonstrated that both EA and AS‐IV effectively improve outcomes in ischemic brain injury (Wang, Chen, et al. 2023; Wang, Liu, et al. 2023). The combination of EA with pharmacological treatments has been shown to surpass the efficacy of drugs alone in certain CNS disorders (Lam Ching et al. 2023; Cao et al. 2020; Liu et al. 2022; Zhong et al. 2022). Our previous findings (Fu et al. 2021) suggested that EA preconditioning at GV20 may induce a brief BBB opening, potentially allowing circulating anti‐inflammatory large molecules to enter and initiate protective immune activation. However, the exact route of their entry remains to be elucidated. Therefore, this study initially aimed to explore whether EA can facilitate the entry of AS‐IV into the brain under intact BBB conditions and to elucidate the underlying mechanisms. On the basis of this, we will further validate the mechanism in the MCAO model. Our findings suggest that a 30‐min EA intervention combined with AS‐IV treatment is more effective in improving behavioral outcomes and reducing infarct size than either treatment alone. This effect extends to the modulation of inflammatory factor levels. Measurement of AS‐IV content in the ischemic hemisphere revealed no significant increase compared to the SHAM + AS‐IV group, indicating limitations in AS‐IV delivery to the brain. EA appears to alleviate this restriction, enhancing AS‐IV content in the ischemic brain. This enhancement may also be linked to EA's potential influence on the systemic pharmacokinetics of AS‐IV, a possibility that warrants further investigation. Further the analysis of MMP‐9 and BBB structural proteins indicated that EA alone did not significantly alter the levels of MMP‐9, ZO‐1, or Claudin‐5. However, AS‐IV and the combination of EA and AS‐IV improved these levels. Notably, although EA combined with AS‐IV did not significantly affect ZO‐1 expression, it significantly increased Claudin‐5 expression, which is consistent with observations under intact BBB conditions. This also suggests that in the ischemic model, EA facilitates AS‐IV entry into the brain via mechanisms other than the paracellular pathway. Evaluation of P‐gp and related proteins revealed no significant alterations in expression levels following EA treatment. Additionally, AS‐IV induced P‐gp expression in the brains of stroke rats, explaining the lack of significant increase in intracerebral AS‐IV levels in the AS‐IV group. However, the addition of EA effectively reduced the elevated P‐gp levels induced by AS‐IV. Furthermore, AS‐IV increased both total and nuclear levels of NF‐κB, whereas EA effectively reversed this increase. EA showed different regulation to AS‐IV‐induced P‐gp expression under varying BBB conditions. This indicates that EA's regulation to AS‐IV‐induced P‐gp via NF‐κB inhibition exhibits dynamic scope. Within a normal BBB, it confined to blocking NF‐κB nuclear translocation. However, under ischemic conditions, its effect expands to suppress the pathological induction of NF‐κB synthesis and activation. This shift from fine‐tuning to comprehensive suppression highlights EA's multi‐tiered regulatory capacity in maintaining BBB homeostasis. However, the limitation of this study is that we did not explore the upstream regulators of NF‐κB, such as IKK phosphorylation, which will be a focus of our future studies.

This work lays a foundation for understanding the synergistic strategy, and future research building upon these findings could address additional informative questions. For instance, it would be informative to include relevant positive control to strengthen the mechanistic interpretation. Additionally, evaluating the specificity of GV20 by comparisons with non‐acupoints or other acupoints would be a valuable next step. These focused investigations will be crucial for comprehensively clarifying the strategy's precise mechanism and clinical promise.

## 5 Conclusion

The combination of EA and AS‐IV enhances the physical barrier of BBB in physiological situations, potentially mediated by β‐catenin/GSK‐3β. Moreover, irrespective of BBB integrity, EA reduces P‐gp expression by inhibiting NF‐κB nuclear translocation, thereby facilitating AS‐IV entry into the brain and enhancing its neuroprotective effects. These findings highlight EA as a promising intervention for promoting the delivery of neuroprotective agents to the CNS.

## Author Contributions

**Ling Ouyang**: conceptualization, methodology, data curation, writing – original draft, formal analysis, investigation. **Xinyi Yang**: methodology, validation, writing – original draft, data curation, software. **Jiayue Wu**: methodology, validation, project administration, investigation. **Bufan Wu**: methodology, validation, project administration, investigation. **Qidong Huang**: methodology, software, resources. **Yonglin Chen**: methodology, software, formal analysis. **Xiaolong Zhang**: investigation, validation, project administration. **Yi Cao**: investigation, validation, project administration. **Ying Chen**: investigation, validation, project administration. **Yirong Yang**: investigation, validation, project administration. **Jingjing Zhang**: investigation, validation, project administration. **Xiaobei Hao**: supervision, resources, formal analysis. **Shengfeng Lu**: supervision, resources, formal analysis. **Xinyue Jing**: conceptualization, funding acquisition, writing – review and editing, supervision, resources. **Shuping Fu**: conceptualization, funding acquisition, writing – review and editing, supervision, resources.

## Funding

This work was supported by the National Natural Science Foundation of China (No. 81774403), the Traditional Chinese Medicine Technology Development Project of Jiangsu Province (No. ZD202304), the Key University Science Research Project of Jiangsu Province (Nos. 22KJA360003 and 22KJA360005), and the Open Projects of the Discipline of Nursing of the Nanjing University of Chinese Medicine Supported by the Subject of Academic Priority Discipline of Jiangsu Higher Education Institutions (YSHL202542).

## Ethics Statement

All animal experimentation protocols were approved by the Institutional Animal Care and Use Committee of the Nanjing University of Chinese Medicine (SCXK(HU) 2022‐0004).

## Conflicts of Interest

The authors declare no conflicts of interest.

## Supporting information




**Supplementary Figure S1**: brb371134‐sup‐0001‐figureS1.docx

## Data Availability

The data sets used and/or analyzed during the current study are available from the corresponding author upon reasonable request.
