# Supplementary material for: Electroacupuncture Potentiates Astragaloside IV Cerebral Delivery via P‐Glycoprotein‐Mediated Transcellular Transport: A Novel Blood–Brain Barrier Penetration Strategy for Ischemic Stroke Therapy
Source: Brain Behav. 2025 Dec 17;15(12):e71134. doi: 10.1002/brb3.71134 (PMC12710072; doi:10.1002/brb3.71134)
Supplement: Supplementary file 1 — Supplementary Figure S1: brb371134‐sup‐0001‐figureS1.docx [file BRB3-15-e71134-s001.docx]

**Supplementary Figure**


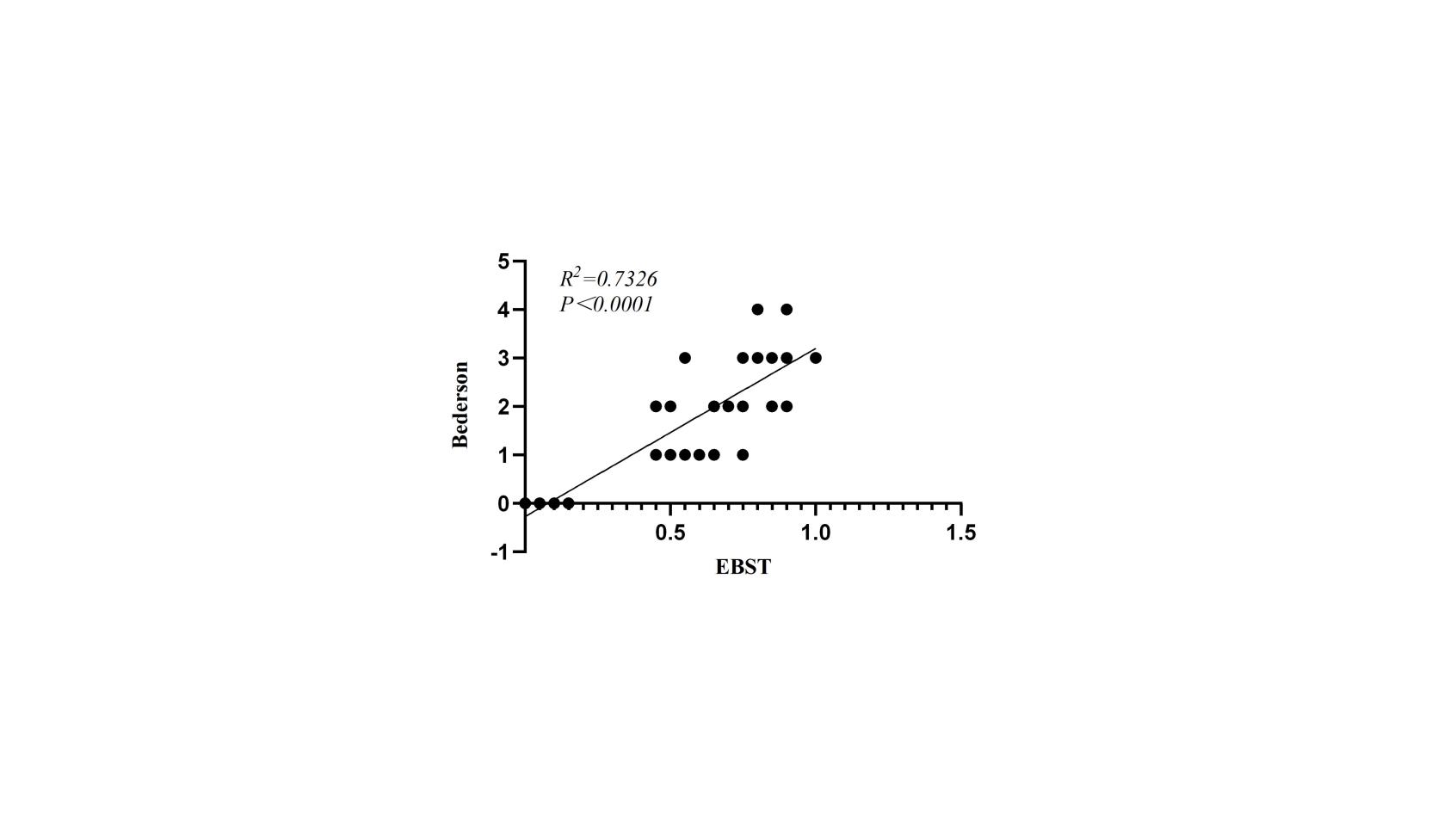


**SUPPLEMENTARY FIGURE 1** Correlation analysis between the Bederson score and the EBST. R^2^=0.7326, P<0.0001.
